# Conventional and synchronous spectrofluorometric determination of the recently administered drugs for treatment of COVID-19 favipiravir and apixaban

**DOI:** 10.1038/s41598-022-25917-5

**Published:** 2022-12-13

**Authors:** Heba Samir Elama, Abdallah M. Zeid, Shereen M. Shalan, Yasser El-Shabrawy, Manal I. Eid

**Affiliations:** Pharmaceutical Analytical Chemistry Department, Faculty of Pharmacy, Mansoura University, Mansoura, 35516 Egypt

**Keywords:** Analytical chemistry, Chemistry

## Abstract

COVID-19 is a fast-spreading pandemic that is caused by SARS-CoV-2 viral pathogen. Combination therapy of the antiviral favipiravir and the anticoagulant apixaban is one of the efficient treatment regimens. Therefore, development of novel and sensitive methods for simultaneous analysis of such combination is highly advantageous. Herein, two eco-friendly, simple, rapid, and cost-effective spectrofluorometric methods were evolved for the estimation of favipiravir and apixaban in pharmaceutical and biological matrices. Method I was based on analysis of favipiravir and apixaban by the first-order derivative of the conventional fluorescence spectra obtained after excitation at 300 nm, where favipiravir and apixaban were detected at 468.8 and 432.0 nm, respectively. Method II relied on dual scan synchronous spectrofluorometry, in which favipiravir was determined at 364 nm using Δλ = 60 nm while apixaban was analyzed at 274 nm using Δλ = 200 nm. Method optimization was performed for selecting the optimum conditions at which maximum sensitivity and selectivity were obtained. This report is the first one that describes simultaneous analysis of favipiravir and apixaban by synchronous spectrofluorometry. The developed methods were successfully applied to evaluate favipiravir and apixaban in spiked human plasma and in pharmaceutical dosages with high %recoveries and low RSD.

## Introduction

COVID-19 pandemic emerged in Wuhan, China at the end of 2019. Up to date, more than 6 million people have lost their lives because of this epidemic out of approximately 492 million who were primarily infected worldwide^1^. The disease is caused by novel infectious positive single-stranded RNA (SARS‐CoV‐2) virus, and it is usually accompanied by multiple atypical pneumonia. Moreover, it shows several other symptoms such as cough, fever, fatigue, diarrhea and progressing to acute respiratory distress syndrome (ARDS). COVID-19 patients may also report neurological symptoms, including dizziness, hypogeusia headache, myalgia, ataxia, hyposmia, and seizures^2^.

Notwithstanding, COVID-19 treatment protocols are still under investigation and may differ from a country to another relying to severity and spread factors, however, the main treatment guidelines are followed. Because of the high reproductive number of SARS-CoV-2, antivirals were tested for efficacy to control or even stop the viral transmission. Antivirals that inhibit reverse transcription are among the most potent agents available to combat the SARS-CoV-2 infection^3^. Among these effective antivirals is favipiravir (FAV) which was recently used for treatment of COVID-19 in some countries.

Favipiravir is a nucleoside analogue (purine base) that is converted intracellularly to the active form, FAV-ribofuranosyl-5B-triphosphate by phosphoribosylation process. It inhibits RNA-dependent RNA polymerase (a core enzyme in SARS-CoV-2 replication process) selectively and potently in SARS-CoV-2. FAV was the antiviral of choice for the current study as it showed rapid viral clearance in comparison to ritonavir and lopinavir in addition to superior rate of recovery compared to umifenovir. Additionally, FAV is incorporated recently in COVID-19 treatment protocols in some countries such as India^4^.

The mortality rate increased in COVID-19 patients who suffered from multi-organ dysfunction accompanying ARDS^5^, elevated D-dimer levels and prothrombotic parameters. Then, anticoagulants were included as prophylactic treatment in patients with high D-dimer values to reduce venous thromboembolism rate or mortality. Reported clinical trials assured the claim by significantly decreasing mortality upon using anticoagulant therapy among COVID-19 protocol^6^. Different anticoagulants were tested for decreasing mortality, enoxaparin and heparin therapeutic doses have not shown to downside mortality rates while apixaban (APX) prophylaxis and therapeutic doses have done. Then, APX was the preferred anticoagulant regimen^6^.

**Figure 1:**
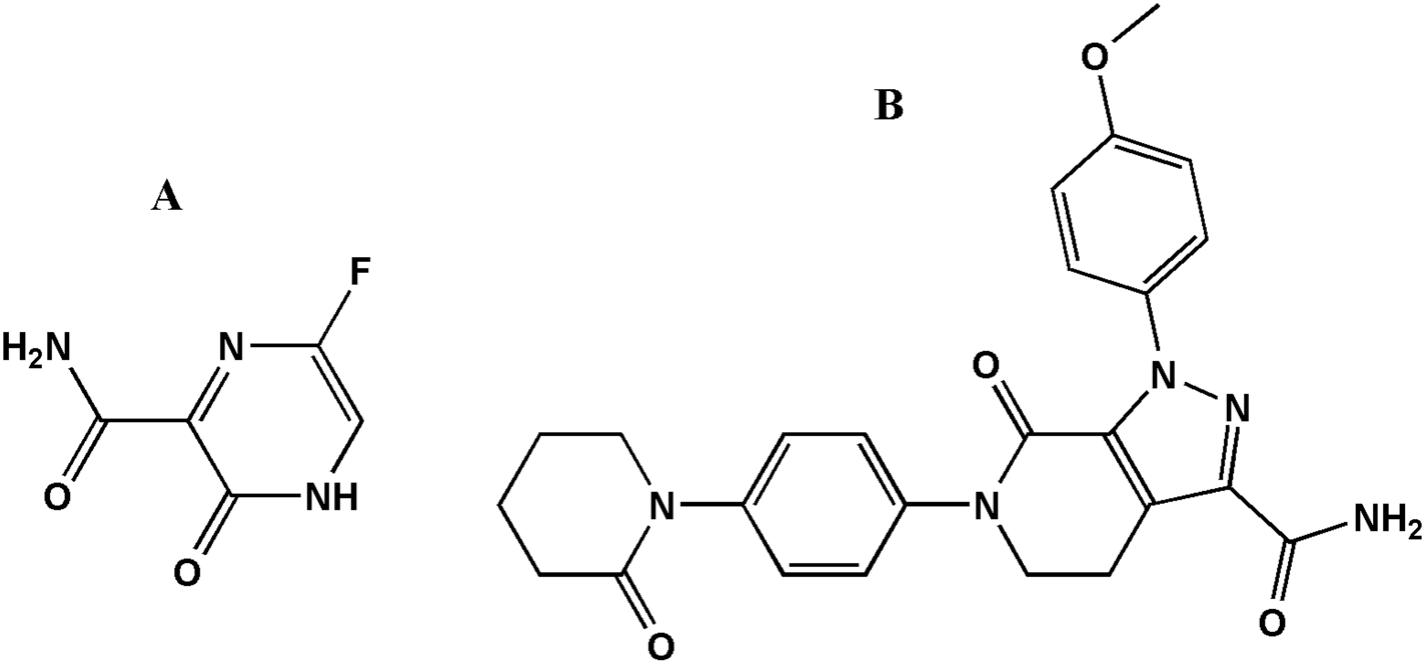
Chemical structures of (**A**) Favipiravir and (**B**) Apixaban.

Favipiravir and apixaban (Fig. 1) have the chemical names of 5-fluoro-2-oxo-1*H*-pyrazine-3-carboxamide and 1-(4-methoxyphenyl)-7-oxo-6-[4-(2-oxopiperidin-1-yl)phenyl]-4,5-dihydropyrazolo[3,4-c]pyridine-3-carboxamide, respectively.

The literature was screened for analytical procedures that were applied for determination of FAV and APX. For FAV, a spectrophotometric method^7^, two conventional spectrofluorometric methods^8,9^, electrochemical methods^10–12^, and HPLC methods^7,9,13^ were reported. On the other hand, spectrophotometric^14–17^, spectrofluorometric^18^, electrochemical^19^, and HPLC^20–24^ methods were reported for the assay of APX. As discussed, antivirals besides anticoagulants are co-administered during COVID-19 treatment in mild to severe cases of infection. The analytical study of such coadministration besides few reported spectrofluorimetric techniques were the motivation to develop this work. It is noteworthy that this study is the first to be performed targeting simultaneous determination of FAV and APX followed by application to human biological samples.

Based on the native fluorescence of FAV and APX, trials for separation of FAV and APX’s overlapped spectra were performed. Then, the two current methods were developed and applied to local market tablets having FAV or APX as the main active ingredient besides determination of both drugs simultaneously in human plasma samples.

## Methods

### Instruments

All spectrofluorometric measurements were performed by an Agilent® Cary Eclipse spectrofluorometer to which a xenon flash lamp is equipped. A smoothing factor of 20, a 5 nm slit width, and an applied voltage of 800 V were used during all experimental trials. Relative fluorescence intensities (RFI) of the estimated analytes were measured using excitation wavelength of 300 nm in method (I) and dual scan in method (II), where in first scan FAV was determined using Δλ of 60 nm and calibrated at 364 nm and in the second scan Δλ of 200 was set to detect APX and then calibration was performed at 274 nm.

### Reagents and materials

Favipiravir raw material was kindly supplied by EIPICO pharmaceutical company, Egypt. Avipiravir® tablets (lot. No. 2008230; 200 mg FAV per tablet) were supplied by EVA Pharma Company, Cairo, Egypt. Apixaban raw material was supplied by Multi Apex company, Egypt. Artixiban® commercial tablets, labelled to contain 2.5 mg APX per tablet, were purchased from a local pharmacy.

HPLC grade iso-propanol, acetonitrile, ethanol, methanol, and n-propanol were all obtained from Sigma-Aldrich, Egypt. Analytical grade of surfactants: tween 80, sodium dodecyl sulfate, and carboxymethyl cellulose were supplied by laboratories of faculty of pharmacy, Mansoura University, Egypt.

The frozen plasma samples were supplied by Hospitals of Mansoura University, Dakahleya, Egypt. All experiments were performed according to the Institutional Ethics Approval of the relevant University Committee.

### FAV and APX standard solutions

Stock solutions, having the concentration of 100 μg mL^−1^ of both drugs, were prepared by accurately weighing 10 mg of the raw materials, quantitatively transferring each powder to a 100 mL volumetric flask, and completing the volume to 100 mL using ethanol, followed by a 10-min sonication process.

### Practical procedures

#### Method I

Sets of 10.0 mL volumetric flasks were arranged, to which aliquots of FAV or APX or both (as in synthetic mixture) were transferred. All flasks were completed to 10.0 mL using distilled water and mixed well. Against blank distilled water, RFI of flasks were measured setting excitation wavelength of 300 nm. The recorded emission spectra were converted to their first order derivatives using filter size of 20 and interval of 5. FAV was measured at 468.8 nm which represented a clear zero crossing point for APX, meanwhile, APX measurements were recorded at 432 nm, that was a clear zero crossing point for FAV. Then, calibration curves were attained by plotting ∆F of each flask against its corresponding concentration.

#### Method II

FAV calibration curves. A set of 10.0 mL volumetric flasks were arranged, to which aliquots of a diluted stock (100 ng mL^−1^) were transferred. Flasks were completed with distilled water to the mark and mixed well. RFI of flasks were recorded at Δλ = 60 nm and calibrated at 364 nm against blank. Calibration curves were attained by plotting ∆F of each flask against its corresponding concentration.

#### APX calibration graphs

Another set of 10.0 mL volumetric flasks were arranged, to which APX aliquots were transferred. Flasks were completed with distilled water to the mark and mixed well. RFI of flasks were recorded at Δλ = 200 nm and calibrated at 274 nm against distilled water as blank. APX calibration curves were attained by plotting ∆F value of each flask against its corresponding concentration.

### Methods application

#### FAV-APX synthetic mixtures

Considering FAV and APX concentration ranges stated in Table 1, aliquots of FAV and APX stock solutions were quantitatively put into 10-mL volumetric flasks series, filled to the mark with distilled water, and mixed well. Mixed flasks were subjected to method (I) and (II) procedures. Method (I): flasks emission RFI were recorded setting excitation wavelength of 300 nm. Then, the recorded emission spectra were converted to their first order derivatives, where FAV was measured at 468.8 nm and APX measurements were recorded at 432 nm. Method (II): dual scan measurements were applied. At first scan, FAV synchronous peaks were recorded at 364 nm using Δλ = 60 nm without overlap with APX. At second scan, APX synchronous spectra were performed at Δλ = 200 and calibrated at 274 nm with no interference with FAV at the selected wavelength. % Recoveries were calculated using the preset calibration graphs or their corresponding regression equations.

**Table 1:** Obtained data for the proposed spectrofluorimetric methods.

Method	I		II	
Parameter	FAV	APX	FAV	APX
Concentration Range (ng mL^−1^)	10–100	100–1000	1–14	100–2000
LOD (ng mL^−1^)	4.79	10.0	0.07	2.07
LOQ (ng mL^−1^)	10.0	30.0	0.22	6.29
Correlation coefficient (r)	0.9999	0.9998	0.9999	0.9999
Intercept	− 2.79	0.53	1.10	7.88
Slope	− 0.48	30.10	64.75	0.313
S_y/x_*	1.43	0.34	6.00	0.82
S_a_**	0.69	0.11	1.46	0.20
S_b_***	0.02	0.44	0.51	5.20 × 10^–4^
% Error	0.41	0.45	0.46	0.24
%RSD	1.01	1.18	1.20	0.58
Mean found (%)	99.98	99.69	100.81	100.20
± S.D	1.01	1.18	1.21	0.59

*Standard deviation of the residuals.

**Standard deviation of the intercept of regression line.

***Standard deviation of the slope of regression line.

#### Avipiravir® and Artixiban® average content determination

For this application, commercial tablets were obtained. Contents of 10 identical tablets were mixed well by mortar trituration. Accurate amounts of tablets powder equivalent to 10.0 mg FAV and 2.5 mg APX were transferred into 100 mL and 25 mL volumetric flasks, respectively. About half volume of flasks were filled with ethanol, then the flasks were sonicated for 30 min and then completed to final marks with ethanol. The solution in each flask was then filtered in separate flasks discarding first few milliliters. Further dilutions were performed with distilled water in order to obtain working standard solutions to be analyzed following the discussed procedures for avipiravir® and artixiban®, in methods (I) and (II), respectively. The nominal contents were calculated using corresponding regression equations or already plotted calibration graphs for each method.

#### Plasma samples spiked with FAV-APX mixture

The donated plasma was kept frozen at − 5 °C then gently thawed in a water bath at 37 °C before using. Into a set of 6 centrifugation tubes, 15-mL each, 1 mL of thawed human plasma was transferred to each tube, followed by spiking with elevating concentrations of FAV and APX, simultaneously. The 6-tubed set examined a one-minute vortex mixing and after that, completed to 5 mL with ethanol that allowed one step precipitation of plasma proteins. The vortexed set examined a 20-min 5500 rotations per minute centrifugation to efficiently separate the precipitated plasma proteins while maintaining FAV and APX in the supernatant layer. Then, supernatants were filtered by 0.2 µm disc filters to remove possible suspended particles. A specified volume (1.0 mL) of the supernatant solutions, containing FAV and APX, was transferred to a series of 10.0 mL volumetric flasks, then the procedures under “FAV-APX synthetic mixtures” section were followed and the %recoveries were measured.

### Ethics declarations

Plasma application was licensed by Research Ethics Committee, Faculty of Pharmacy, Mansoura University (Code number: 2022-128). The Research Ethics Committee, Faculty of Pharmacy, Mansoura University (Code number 2022-128), waived the need for informed consent to be obtained. Also, all experiments followed the guidelines and regulations by Research Ethics Committee, Faculty of Pharmacy, Mansoura University.

## Results and discussion

COVID-19 or corona disease is still attacking all countries worldwide with variable strengths. Treatment protocols passed through several updates and are still under study and optimization by clinical researchers. Then, we had the eager to develop the presented methods which manifested the selectivity and sensitivity of spectrofluorometric technique. Our methods estimated the recently approved COVID-19 antiviral FAV and co-administered APX anticoagulant in their dosage forms. Additionally, both drugs were determined simultaneously in human plasma samples, and this would be further applied in forensic medicine and clinical studies.

**Figure 2:**
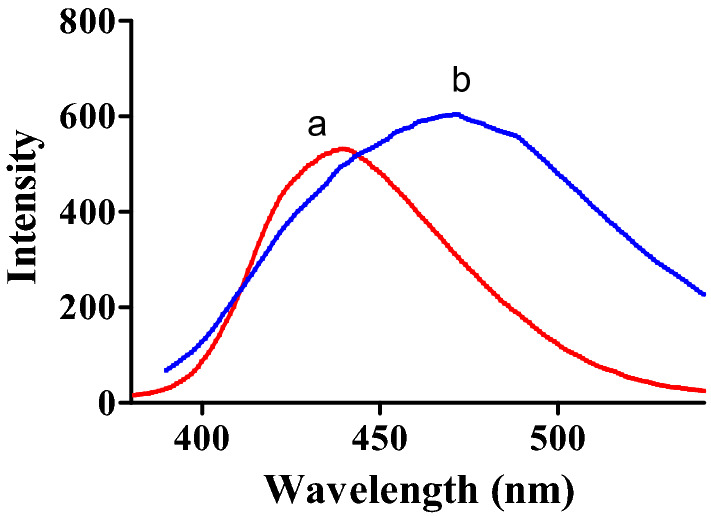
Overlaid overlapped emission spectra of (**a**) FAV, 30.0 ng mL^−1^ after excitation at 323 nm and (**b**) APX 2.0 µg/mL following excitation at 284 nm.

Spectrofluorometry was the technique of choice as it is featured by high sensitivity, selectivity, simplicity, and low cost. This technique allowed utilization of reported conventional fluorescence spectra of the analytes in question^9,18^. Upon spectrofluorometric scanning of FAV and APX, a complete overlap was observed as represented in Fig. 2. Hence, trials to resolve this overlap were carefully conducted.

**Figure 3:**
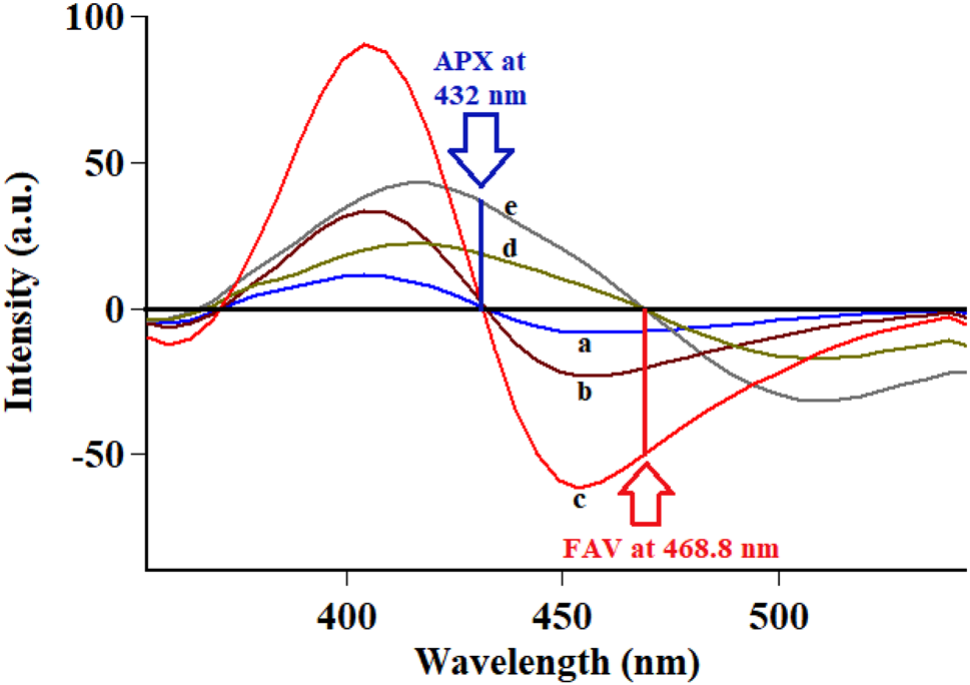
Overlaid first order derivative of emission spectra of FAV (a,b,c in concentrations of 10.0, 25.0 and 100.0 ng mL^−1^) and APX (d and e in concentrations of 0.5 and 1.0 μg mL^−1^) after excitation at 300 nm.

**Figure 4:**
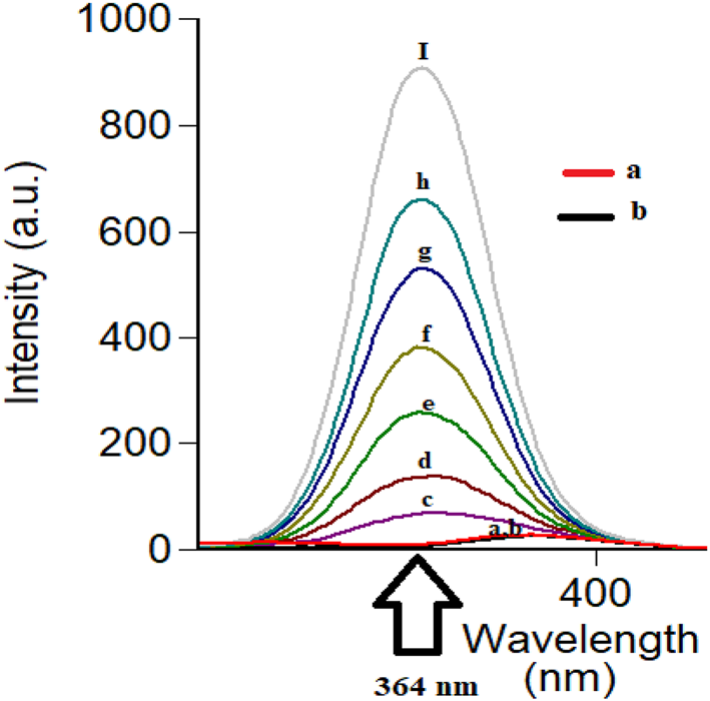
Overlaid synchronous spectra at Δλ of 60 for FAV calibration (**c**–**i**) and blank (**a**) and (**b**) APX 1.0 μg mL^−1^.

**Figure 5:**
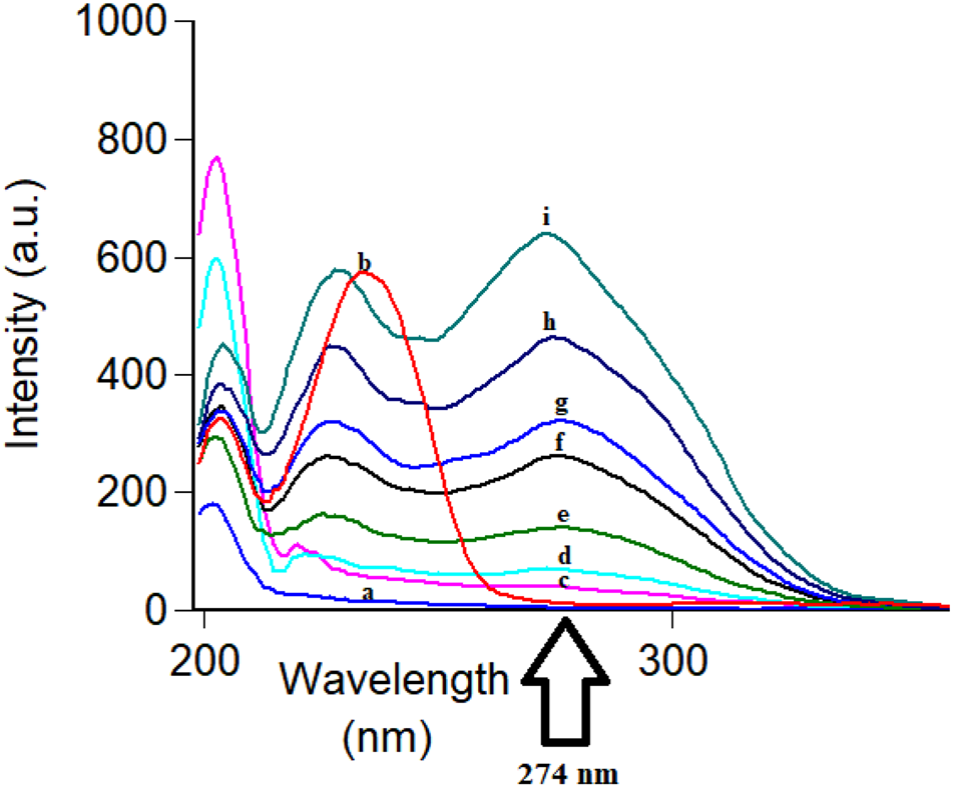
Overlaid synchronous spectra at Δλ of 200 for APX calibration (**c**–**i**) and blank (**a**) and FAV 10.0 ng mL^−1^ (**b**).

Following conventional emission of FAV and APX at their reported λ_ex_ of 323 nm and 248 nm, respectively, an overlap was observed hindering simultaneous measurement of the analytes in question Fig. 2. To resolve the observed overlap, both conventional and synchronous spectrofluorometry were examined as reported means of simultaneous measurements without preliminary sample treatments^25,26^. In conventional spectrofluorometry, recording emission at excitation of 300 nm allowed detection of FAV and APX simultaneously at 468.8 nm and 432 nm, respectively after implementing first order derivatization as in Fig. 3, method (I). Synchronous fluorescence spectrofluorometry (SFS) using constant wavelengths was tried over different Δλ values ranging from 20 to 200 nm. Δλ of 60 was of the highest intensity for FAV at 364 nm and demolished the intensity of APX even in high concentrations as in Fig. 4. While Δλ of 200 exerted higher intensities for APX at 274 nm abolishing FAV intensity at the same wavelength Fig. 5. Trials to adjust an optimum Δλ values in the middle of this wide range was not satisfactory due to dense overlap that was not resolved by first or second order derivatizations. Hence, dual scan was of choice to keep high intensity and selectivity of both drugs to determine each of them in presence of the other one with no overlap.

SFS is advantaged by simplicity and sharpness of peaks when compared to conventional spectra. Beside minimization of complex matrix interference, high selectivity is guaranteed by synchronous technique, then plasma applications are more simple^27,28^.

### Optimization of the study

Spectrofluorometry is characterized by high sensitivity, then study conditions were cautiously examined. Where constant concentrations of FAV (10 ng mL^−1^) and APX (1.0 µg mL^−1^) were used for the following three parameters. Conventional spectrofluorometry was adopted to adjust optimum conditions using emission 432 nm after 323 nm excitation for FAV samples and emission at 475 nm after 280 nm excitation for APX samples.

#### Surfactants

**Figure 6:**
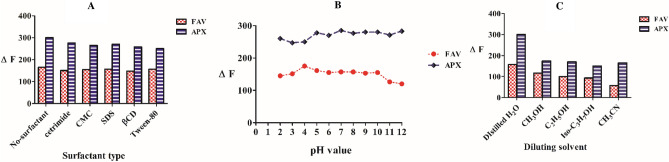
Optimization of method conditions using FAV: 10 ng mL^−1^ at emission 432 nm after 323 nm excitation and APX 1.0 µg/mL at 475 nm after 280 nm excitation.

Different surfactants were examined to detect whether an increase in fluorescence intensity was observed. Figure 6A summarizes the obtained data after trying different surfactants namely cetrimide, carboxymethylcellulose, Na dodecyl sulfate, b-cyclodextrin, and tween 80. All obtained results were compared to the initial no surfactant trials referring that neither FAV not APX showed noticeable enhancement of fluorescence intensity using the examined surfactants.

#### Buffering pH

Solution’s pH may be of a great impact on fluorescence intensity whether elevating or down siding. As pH may affect resonance and conjugation forms of the studied drugs, different pH values were tested for FAV and APX. As shown in Fig. 6B, Britton Robinson buffer systems with pH values ranging from 2 to 12 were examined. The results indicated a clear change in the fluorescence intensity of the studied analytes at the extreme pH values. However, this change is not significant and does not participate in improving the method sensitivity. For instance, at high pH values (e.g., pH 12), APX exhibited high fluorescence intensity, but the other drug (FAV) exhibited a dramatical decrease in its intrinsic fluorescence. In general, it is observed that using the buffer system at a pH range of 4–10 gave acceptable results for both drugs. However, the results obtained without using a buffer system, were not significantly different from these results. That’s why we did not use buffers for the simplicity and time-saving purposes.

#### Diluting solvents

The nature of diluting solvents possesses different effects on fluorescence magnitude of the studied drugs. For instance, energy levels of electrons in π* orbitals and non-bonding electrons may be changed for hydrogen-bonding solvents, then λ_max_ of both emission and excitation spectra may be altered^29^. Then, methanol, ethanol, isopropanol, acetonitrile, and distilled H_2_O as different diluting solvents were under examination. As Fig. 6C depicts, distilled H_2_O showed 60% enhancement of APX fluorescence intensity besides highest magnitude for FAV samples. Having chosen distilled H_2_O as a diluting solvent, method simplicity, environmental friendliness and low cost were guaranteed throughout method application.

#### Selection of excitation wavelength in method I

It was reported that excitation wavelength could be utilized to separate overlapped emission spectra^26^. FAV and APX were reported to have the optimum excitation wavelengths of 323 nm^9^ and 284 nm^18^, respectively. Then stepwise trials of excitation wavelengths were triad ranging from 280 to 320 nm observing emission spectra of both analytes. The excitation wavelength of 300 nm was satisfactory for separation of FAV and APX combination with high sensitivity. For further elimination of spectral overlap, first order mathematical derivatization was implemented with different intervals and filter size values. Filter size of 20.0 and interval of 5.0 showed an ideal peak integration and separation as depicted in Fig. 3.

#### Selection Δλ for dual scan in method II

**Figure 7:**
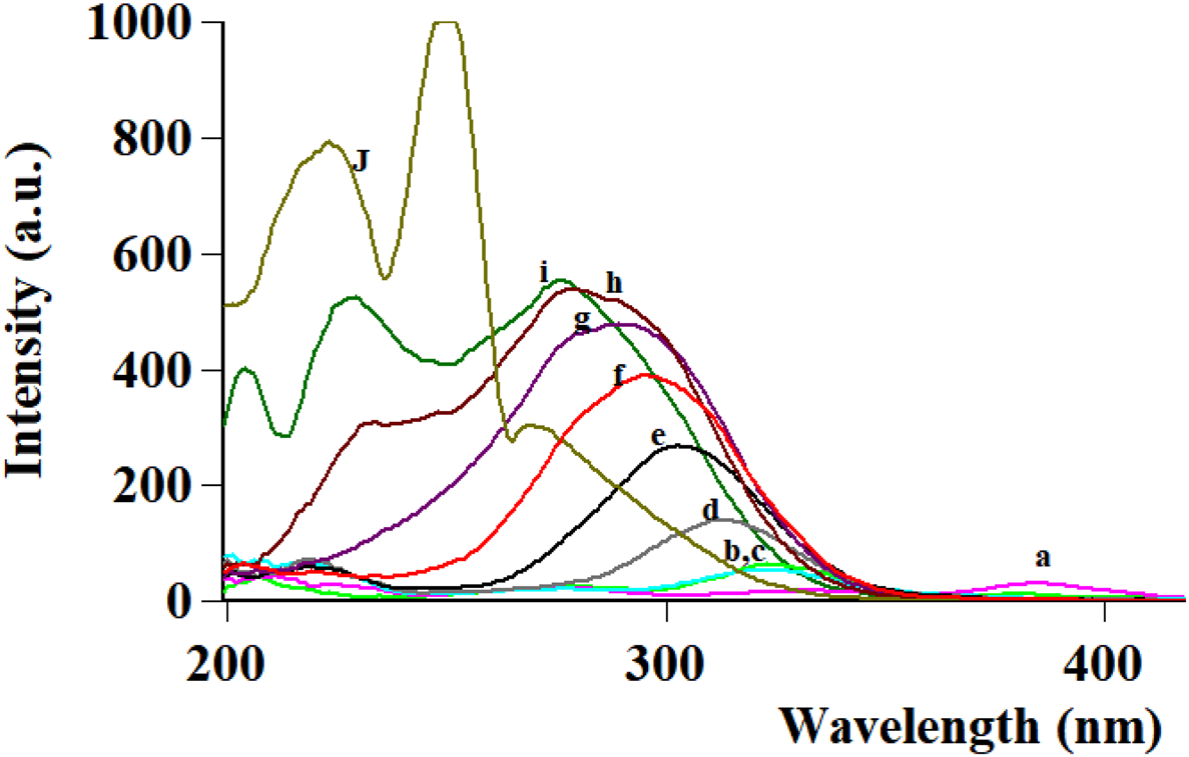
Overlaid stepwise study of different Δλ values for APX, 2.0 µg mL^−1^, where (**a**–**i**), and (**J**) represent Δλ of 40, 60, 80, 100, 120, 140, 160, 180, 200, and 250 nm, respectively.

**Figure 8:**
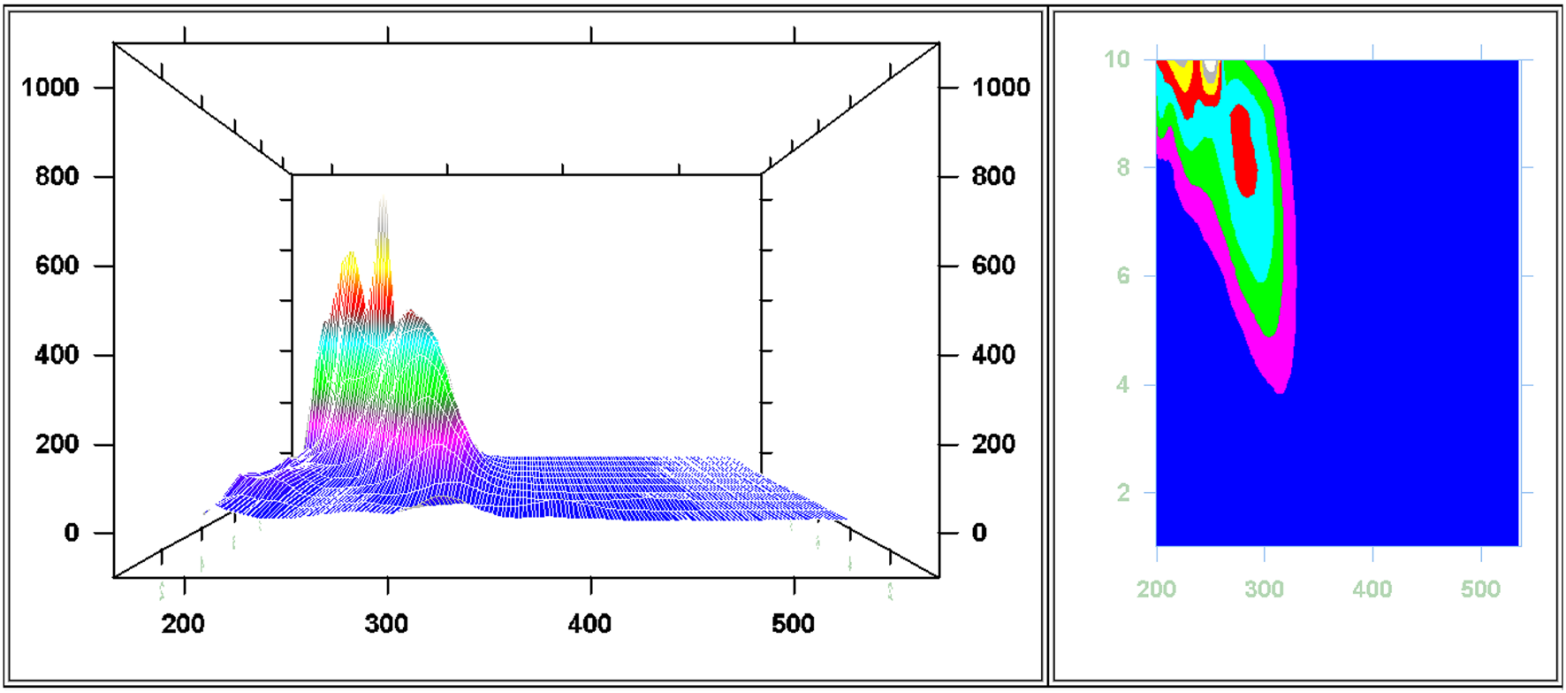
Overlaid 3D and contour designs for stepwise study of different Δλ values for APX, 2.0 µg mL^−1^.

**Figure 9:**
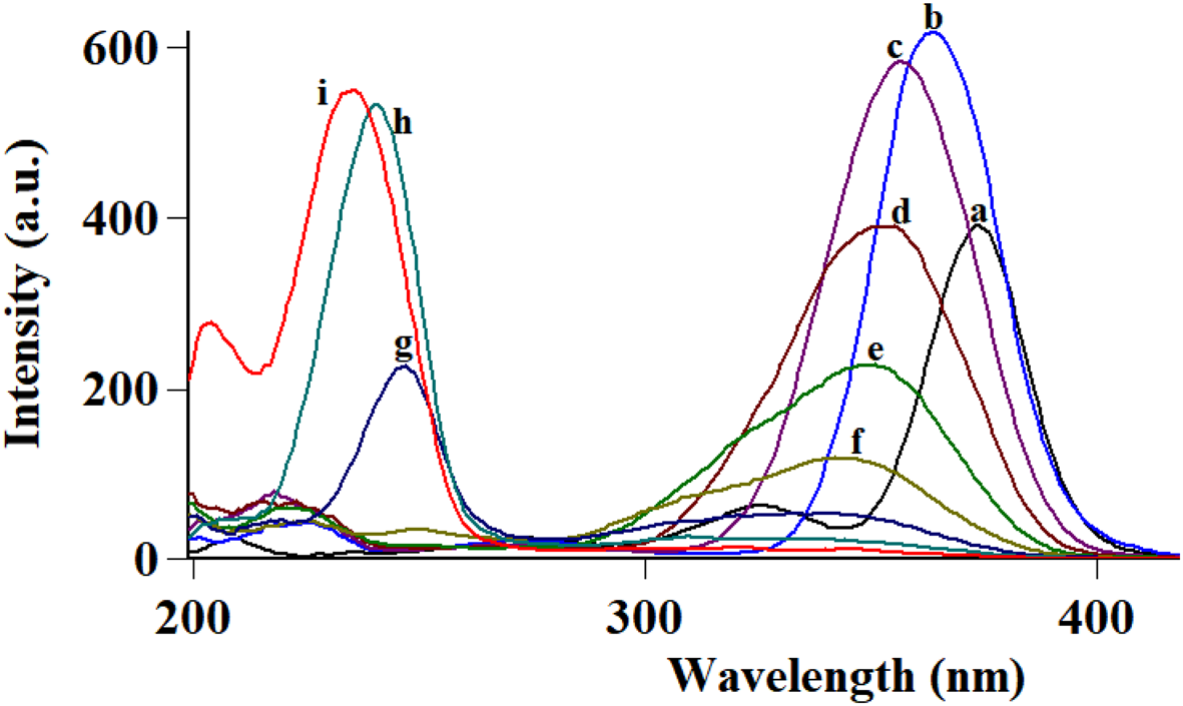
Overlaid stepwise study of different Δλ values for FAV, 10.0 ng mL^−1^, where (**a**–**i**) represent Δλ of 40, 60, 80, 100, 120, 140, 160, 180, and 200 nm, respectively.

**Figure 10:**
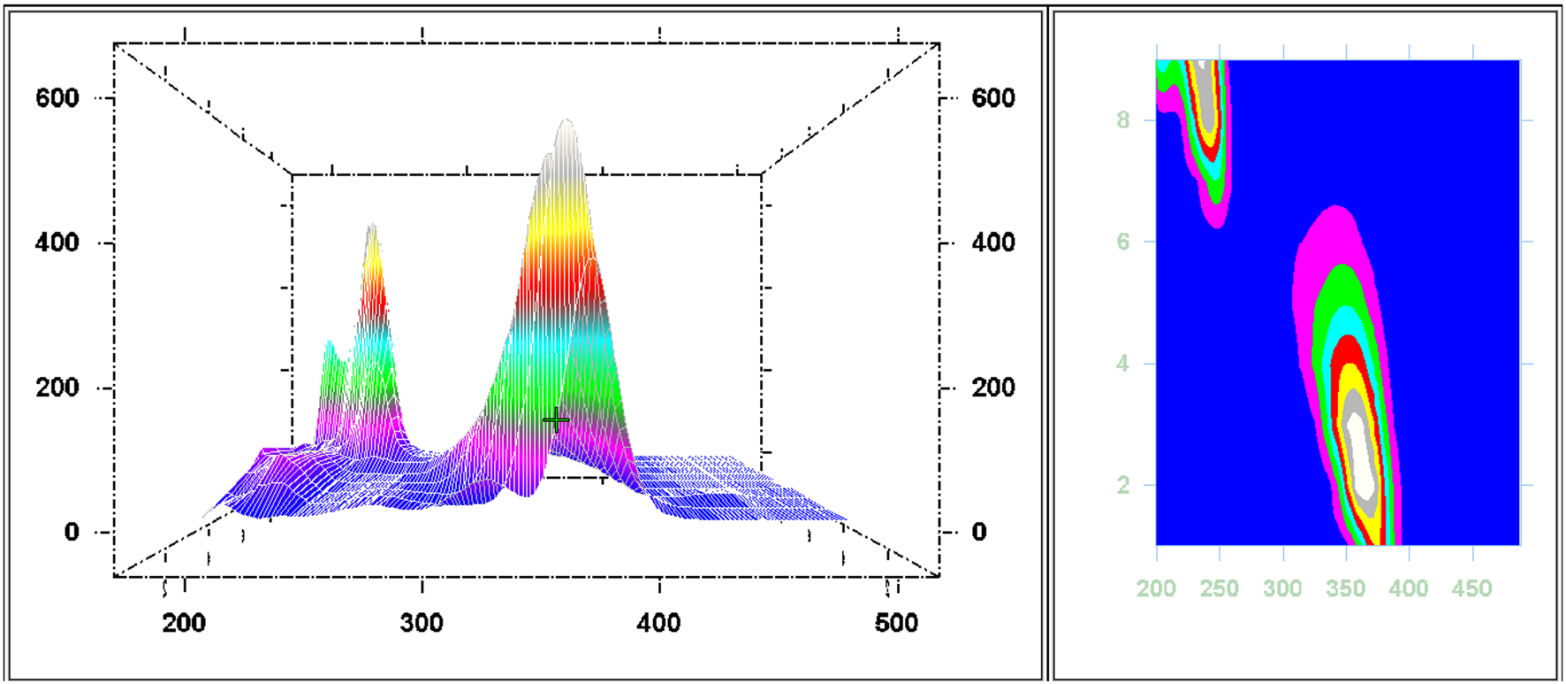
Overlaid 3D and contour designs for stepwise study of different Δλ values for FAV, 10.0 ng mL^−1^.

Following optimum study conditions and owing to impact of Δλ in terms of sensitivity and peak resolution using SFS, different values of Δλ values were tried ranging from 40 to 200 nm for FAV and APX as shown in Figs. 7, 8, 9 and 10. As depicted in the mentioned figures, upon increasing Δλ value, peaks’ maxima were shifted towards Y-axis for both drugs beside appearance of a new peak maxima when Δλ was above 140 nm for FAV.

Three Δλ values were significant for separation; Δλ 60, 160 and 200 nm. First, Δλ 160 was promising as it achieved satisfying sensitivity of both drugs, simultaneously with the least overlap while observing both drugs. Unfortunately, this overlap at Δλ 160 was not resolved using first and second derivative of SFS peaks, then method application was hindered. Second, Δλ 60 was characterized by high intensity and selectivity of FAV at 364 nm due to zero intensity of APX at the same wavelength, so FAV was determined and calibrated efficiently Fig. 4. Third, Δλ 200 exerted highest selectivity of APX at 274 nm with baseline intensity of FAV, at the selected wavelength APX was calibrated successfully with no interference of FAV, Fig. 5. As a result, dual SFS scan was utilized for determination of FAV and APX with enhanced selectivity.

### Method application

#### Average content determination

The proposed method showed successful application to pharmaceutical tablets Avipiravir® and Artixiban® containing FAV and APX, orderly. Following the practical procedure mentioned under “Avipiravir^®^ and Artixiban^®^ average content determination”, the obtained % recoveries and comparing to comparison methods stated as student *t*-test and variance ratio F-test are stated in Table 2. The stated values indicated acceptance of the proposed procedures as calculated student t and variance ratio F tests values lower than the tabulated ones.

**Table 2:** Application of the proposed method to Avipiravir® and Artixiban® commercial tablets.

Parameter	Proposed method (I)		Proposed method (II)		Comparison methods^9,18^
	Amount taken (ng mL^−1^)	% Recovery ^a^	Amount taken (ng mL^−1^)	% Recovery *	% Recovery *
Avipiravir® (Favipiravir, 200 mg/tab)	20	99.37	2	98.62	98.975
	40	99.79	4	98.24	99.13
	60	100.56	6	98.57	100.59
	–	–	–	–	99.89
	–	–	–	–	99.41
Mean ± S.D		99.91 ± 0.60		99.48 ± 0.12	99.60 ± 0.29
*t*		0.66 (2.47) **		2.41(2.47) **	
*F*		1.14 (19.25) **		10.04(19.25) **	
Artixiban® (Apixaban, 2.5 mg/tab)	200	99.92	400	100.00	100.6818
	400	100.75	600	98.00	98.17311
	800	100.64	800	99.13	99.27693
			–	–	101.7566
Mean ± S.D		100.44 ± 0.46		99.04 ± 0.58	99.97 ± 1.57
*t*		0.49 (2.57) **		0.89(2.57) **	
*F*		12.15 (19.16) **		2.46(19.16) **	

*Each result is the average of three separate measurements.

**Tabulated values for *t* and *F* tests.

#### Spiked human plasma

The two spectrofluorometric approaches guaranteed high sensitivity to detect both FAV and APX in human plasma samples spiked by the studied drugs. It is reported that plasma maximum levels in adult humans are 104.7, 176.3, 365.1 and 685.2 ng mL^−1^ following oral single doses of 5, 10, 25, and 50 mg APX, respectively^30^. While FAV is reported to have plasma maximum levels of 4.43 µg/mL in critically ill COVID-19 patients and levels lower than 1 µg mL^−1^ were also stated^31^. Luckily, our methods showed satisfactory mean recoveries for FAV and APX in spiked human plasma samples as shown in Table 3.

**Table 3:** Results for the assay of FAV and APX in human plasma.

Parameter	Spiked plasma samples							
	Method (I)				Method (II)			
	Amount taken (ng mL^−1^)		% Recovery*		Amount taken (ng mL^−1^)		% Recovery*	
Mixture	FAV	APX	FAV	APX	FAV	APX	FAV	APX
	2	200	81.45	99.50	10	200	84.62	112.00
	4	400	116.95	101.00	20	400	106.05	100.25
	7	600	95.51	97.50	40	600	102.76	96.33
	10	800	97.09	102.63	60	800	100.28	98.00
	12	1000	102.17	99.10	80	1000	99.00	102.20
Mean			98.63	99.95			98.54	101.76
± S.D			12.80	1.95			8.23	6.14
Slope			73.32	302.00			− 0.28	39.75
Intercept			− 41.06	12.80			− 0.59	− 3.94
S_y/x_			36.80	7.78			0.65	3.30
S_a_			21.93	5.50			0.38	3.46
S_b_			4.46	12.30			0.01	5.22

*Each result is the average of three individual measurements.

### Method validation

Following ICH guidelines^32^, different validation parameters were estimated. To evaluate methods’ linearity and range, calibration curves were obtained by plotting ΔF values against the corresponding drug concentrations. Linearity ranges were observed over the concentration ranges stated in Table 1. By statistical analysis^33^ of the data obtained, FAV and APX had the following regression formula: method (I), FAV: ΔF = − 2.79 − 0.48C, APX: ΔF = 0.53 + 30.1C , and method (II) FAV: ΔF = 1.10 + 65.29 C, APX: ΔF = 7.88 + 313.22 C, where C: drug concentration and ΔF : relative fluorescence intensity.

Detection and quantitation limits values were both calculated by data obtained in calibration graphs following using the reported equations: LOD = $\frac{3.3\mathrm{ Sa}}{\mathrm{slope}}$, and LOQ = $\frac{10\mathrm{ Sa}}{\mathrm{slope}}$, where LOD is the limit of detection, LOQ is the limit of quantitation, and S_a_ is the standard deviation of intercept. Table 1 summarized values of LOD and LOQ for FAV and APX. Table 1 also states correlation coefficient values obtained by further statistical analysis of FAV and APX calibration graphs. (*r*) of 0.9999 for both drugs assured linearity of methods for both drugs in the selected range of analysis.

Accuracy of the proposed spectrofluorometric procedures could be expressed by accepted values of Student *t*-test and variance ratio F-test when compared to comparison procedures. Obtained data and analysis values are expressed in Table 4.

**Table 4:** Method application to raw materials and synthetic mixtures of FAV and APX compared to their comparison methods.

Compound	Raw materials									
	Proposed method (I)				Proposed method (II)				Comparison methods^9,18^	
	Amount taken (ng mL^−1^)		%Found*		Amount taken (ng mL^−1^)		%Found*		% Found*	
	FAV	APX	FAV	APX	FAV	APX	FAV	APX	FAV	APX
	10	100	100.50	98.00	1.0	100	101.00	100.50	100.32	98.19
	20	200	99.78	99.5	2.0	200	101.85	101.24	99.65	100.82
	40	400	98.37	99.5	4.0	400	101.90	99.88	101.86	99.66
	80	500	100.42	100.6	6.0	800	98.57	99.83	101.86	100.50
	60	800	101.32	101.38	8.0	1000	101.53	99.66	99.25	
	100	1000	99.51	99.10	10.0	2000	100.99	100.11	100.86	
					14.0		99.83			
*t*			1.07(2.23)**	0.15(2.31)**			0.98(2.18)**	0.53(2.31)**		
F			1.18(5.05)**	1.01(6.59)**			1.39(4.28)**	4.83(6.59)**		
**Synthetic mixtures**										
Compound	FAV	APX	FAV	APX	FAV	APX	FAV		APX	
	20	800	101.13	102.12	4.0	200	99.59		101.28	
	40	400	99.41	100.10	6.0	600	100.53		101.54	
	60	1000	98.28	98.39	8.0	1000	100.03		100.89	
Mean ± S.D			99.61 ± 1.44	100.20 ± 1.87			100.05 ± 0.47		101.24 ± 0.33	
S.E			0.83	1.08			0.27		0.19	

*Each result is the average of three separate measurements.

**Tabulated values for t and F tests.

FAV comparison method was based on direct analysis at 436 nm in Britton-Robinson buffer of pH 4 after excitation at 323 nm with linearity range of 20–350 ng mL^−1^^9^. Additionally, the reported comparison method for APX analysis relied on conventional spectrofluorometric measurement at 450 nm following excitation at 284 nm using distilled water with linearity range of 0.2–6.0 μg mL^−1^^18^. The present SFS method is superior in sensitivity for FAV (1–14 ng mL^−1^) and APX (0.1–2.0 μg mL^−1^).

Table 5 recaps the practically obtained inter-day and intra-day precision results for the proposed method. Low values of % RSD and % error indicated reasonable intra and inter-day precision.

**Table 5:** Precision data for the assay of FAV and APX in their raw materials.

FAV			APX		
Sample concentration (ng mL^−1^)	% Recovery^a^ (Intraday precision)	% Recovery* (inter-day precision)	Sample concentration (μg mL^−1^)	% Recovery* (Intraday precision)	% Recovery* (inter-day precision)
4	99.52	99.14	0.4	100.03	98.28
	98.37	97.91		100.65	99.85
	101.82	99.91		100.65	101.45
X′ ± S.D	99.88 ± 1.76	98.98 ± 1.00	X′ ± S.D	100.45 ± 0.36	99.85 ± 1.60
% RSD	1.76	1.01	% RSD	0.36	1.60
% Error	1.02	0.59	% Error	0.21	0.92
6	98.39	98.00	0.6	98.93	98.23
	98.11	97.88		99.03	98.50
	98.51	98.26		99.57	99.03
X′ ± S.D	98.34 ± 0.21	98.05 ± 0.19	X′ ± S.D	99.18 ± 0.34	98.58 ± 0.41
% RSD	0.21	0.20	% RSD	0.35	0.41
% Error	0.12	0.11	% Error	0.20	0.24
8	100.12	99.73	0.8	99.93	100.61
	99.93	99.93		99.66	100.7
	99.54	100.88		101.01	101.15
X′ ± S.D	99.86 ± 0.30	100.18 ± 0.61	X′ ± S.D	100.20 ± 0.71	100.82 ± 0.29
% RSD	0.30	0.61	% RSD	0.71	0.29
% Error	0.17	0.35	% Error	0.41	0.17

*Each result is the average of three individual measurements.

## Conclusions

Two rapid, simple, direct, green, and sensitive spectrofluorometric methods were evolved for the simultaneous analysis of COVID-19 medication regimen FAV and APX in different matrices. The assay methods utilized both conventional and dual scan synchronous fluorescence spectroscopy in order to assay each analyte in presence of the other one without any interference. Dual scan technique has overcome the sensitivity and selectivity problems by measuring each analyte at its λ_max_ in each individual scan. The methods were applied successfully to assay the analytes of interest in their pharmaceutical dosages. Additionally, FAV and APX were analyzed in human plasma with % mean recoveries of 98.54% and 101.76% in conventional spectrofluorometric method and 98.63% and 99.95% in dual scan synchronous spectrofluorometry for FAV and APX, respectively.

## Data Availability

The datasets generated and/or analysed during the current study are available from the corresponding author upon a reasonable request.
